# Caries detection with tooth surface segmentation on intraoral photographic images using deep learning

**DOI:** 10.1186/s12903-022-02589-1

**Published:** 2022-12-07

**Authors:** Eun Young Park, Hyeonrae Cho, Sohee Kang, Sungmoon Jeong, Eun-Kyong Kim

**Affiliations:** 1 Department of Dentistry, College of Medicine, Yeungnam University, Daegu, South Korea; 2 Research Center for Artificial Intelligence in Medicine, Kyungpook National University Hospital, Daegu, South Korea; 3 School of Electronics Engineering, College of IT Engineering, Kyungpook National University, Daegu, South Korea; 4 Department of Medical Informatics, School of Medicine, Kyungpook National University, Daegu, South Korea; 5 Department of Dental Hygiene, College of Science and Technology, Kyungpook National University, 2559 Gyeongsangde-ro, Sangju, Gyeongsangbuk-do South Korea

**Keywords:** Artificial intelligence, Caries localisation, Convolutional neural network, Deep learning, Intraoral camera, Tooth surface segmentation

## Abstract

**Background:**

Intraoral photographic images are helpful in the clinical diagnosis of caries. Moreover, the application of artificial intelligence to these images has been attempted consistently. This study aimed to evaluate a deep learning algorithm for caries detection through the segmentation of the tooth surface using these images.

**Methods:**

In this prospective study, 2348 in-house intraoral photographic images were collected from 445 participants using a professional intraoral camera at a dental clinic in a university medical centre from October 2020 to December 2021. Images were randomly assigned to training (1638), validation (410), and test (300) datasets. For image segmentation of the tooth surface, classification, and localisation of caries, convolutional neural networks (CNN), namely U-Net, ResNet-18, and Faster R-CNN, were applied.

**Results:**

For the classification algorithm for caries images, the accuracy and area under the receiver operating characteristic curve were improved to 0.813 and 0.837 from 0.758 to 0.731, respectively, through segmentation of the tooth surface using CNN. Localisation algorithm for carious lesions after segmentation of the tooth area also showed improved performance. For example, sensitivity and average precision improved from 0.890 to 0.889 to 0.865 and 0.868, respectively.

**Conclusion:**

The deep learning model with segmentation of the tooth surface is promising for caries detection on photographic images from an intraoral camera. This may be an aided diagnostic method for caries with the advantages of being time and cost-saving.

## Background

Dental caries is one of the most common infectious diseases globally that can cause oral pain, infection, and even tooth loss without proper treatment [1–3]. Untreated caries can be a biological, social, and financial burden both for the individual and society as a whole [4]. However, it is usually not life-threatening; therefore, many patients visit the dental clinic for treatment when the caries are at an advanced stage and serious complications, which are expensive or difficult to treat, have already occurred.

Early detection of dental caries reduces the treatment costs and time. It also conforms to the concept of minimally invasive dentistry by avoiding aggressive treatments, such as root canal treatment or tooth extraction [5]. Thus, the early detection of dental caries is required, and this warrants a method that could accurately, easily, and rapidly diagnose dental caries at an early stage.

Conventional examination for caries detection is primarily performed by visual inspection, tactile sensation, and radiography [6], which are clinical evaluations. However, oral health factors are unequal worldwide, and there are still many people in poor condition with limited availability and accessibility to dental professionals [4]. Therefore, there is a need to develop a more convenient and cost-effective technique for early detection of carious lesions. Moreover, there is variability in the ability of dentists to detect caries; therefore, it would be good if a standardised diagnostic method could assist the practitioners [7].

In the field of dentistry, novel techniques beneficial for patients and clinicians are being developed. One such device is an intraoral camera (IOC), which is inexpensive, easy to operate with digital storage, and capable of capturing high-quality images [8, 9]. It has also been shown that caries can be identified simply and reliably using the IOC [10, 11]. Recently, some studies have suggested the application of deep learning algorithms in the diagnosis of caries using oral photographic images [12, 13].

Deep learning (DL) is a subset of machine learning in artificial intelligence (AI) and is based on deep neural networks that consist of multiple hidden layers to progressively learn representations from raw data. In DL, a convolutional neural network (CNN or ConvNet) has demonstrated excellent performance in computer vision and has been most commonly applied to analyse visual imagery [14, 15]. Recently, CNNs have been rapidly emerging in the medical field and have demonstrated excellent performance in computer vision, including object, facial, and activity recognition, tracking, and three-dimensional mapping and localisation [16]. In the dental field, several studies have applied CNNs to detect carious lesions on periapical radiographs [17], radiovisiography [18], and oral photographs [19, 20].

The detection of dental caries from intraoral photographs using DL is inexpensive and accessible, allowing advanced oral healthcare conditions. Furthermore, this computer-aided diagnostic (CAD) technique is a reliable and standardised assistant. However, studies on CNN models for the detection of dental caries in oral photographs are still limited. Zhang et al. showed that DL using oral photographs captured with smartphones was useful for screening dental caries. However, some oral areas are difficult to capture owing to their location; therefore, smartphones are not appropriate for clinical use in professional dental diagnosis [19]. Kühnisch et al. demonstrated the high accuracy of a CNN model, using oral photographs including only one tooth captured by a digital single-lens reflex (DSLR) camera, in caries detection. Also, the photographs were strictly selected according to methodological requirements, and the images similar to caries, such as tooth discoloration or abrasion, were excluded from the datasets; therefore, limitations in daily use in dental clinics may be present [20]. However, relatively large area including several teeth can be captured in one intraoral photograph frequently, which may affect accuracy of CNN model. In case of dental panoramic images, there were some trials to detect carious lesion better by focusing tooth surfaces through image preprocessing with segmentation [21]. Segmentation task by CNN is a kind of classification at every pixel of the input image and can discriminate different anatomical structures in medical images [22]. However, in case of intraoral photograph, there was little trials to separate tooth area from background by segmentation for caries detection. Therefore, it is necessary to develop CNN algorithms to detect caries among photographs including various clinical conditions such as developmental tooth defect, discoloration, or dental restorations which can be obtained in dental clinics generally along with applying segmentation for tooth surfaces to improve accuracy.

Finally we evaluated the performance of the CNN algorithms in the detection of dental caries using photograph, which can generally be taken by IOC in dental clinics. Moreover, to improve the performance in the detection of caries, we pre-processed the photographic images by segmenting the tooth surface using the CNN algorithm.

## Methods

### Study protocol and dataset

The study procedure was approved by the Institutional Review Board of the Kyungpook National University (KNU-2021-0097) and was performed in accordance with the Declaration of Helsinki. This study followed the guidelines of the Standards for Reporting of Diagnostic Accuracy Studies (STARD). Participants were recruited from patients who visited the dental clinic of the university hospital, after explaining the purpose and process of the study according to the approved study protocol, from October 2020 to December 2021. Among those, patients who have at least one permanent tooth, and could cooperate with taking photographs using an intraoral camera were selected, and finally 445 patients who provided written informed consents were enrolled. A total of 2348 RGB intraoral photographic images were taken by an experienced dentist using a professional intraoral camera (Qraypen, AIOBIO, Seoul, Republic of Korea) after oral examination. For oral examination, dental mirror and explorer were used carefully under dental light in a unit chair with an air syringe by three dentists. Photographs which have poor image quality for dentist to diagnose and make ground truth, such as blurred, unintended, or duplicated images, were not included in dataset. However, to include general photographic images that can be normally acquired at general dental clinics, there was no criteria for the number of teeth or amount of saliva in the photographic image if the diagnosis of caries was possible. Similarly, various non-carious defects, such as tooth stain, hypomineralisation, tooth wear, or dental restoration, were included in the dataset. All images with a resolution of 1280 × 720 pixels were used for analysis without additional processing.

### Annotation for tooth surface and caries

Each photographic image was examined to detect carious lesion on a personal computer monitor by a board-certified dentist, who is an expert in the epidemiological oral examination conducted by the government agency in Korea. First, all tooth surfaces in each image were labelled pixel-wise by the dentist using a tagging tool (https://supervise.ly) as a reference standard for tooth surface segmentation. Second, ground truth for carious lesion was made on tooth surface using bounding box according to International Caries Detection and Assessment System (ICDAS) considering each clinical chart record [23]. If the carious lesion was separated by a sound tooth surface on one tooth, each bounding box was created independently using the tagging tool mentioned above. Therefore, according to the carious lesion, each photographic image had none or more than one bounding box. To minimise ambiguousness of caries diagnosis using photographic images only, distinct caries classified as codes 4, 5, or 6 according to the ICDAS (ICDAS Code 4: An underlying dark shadow from dentin with or without localised enamel breakdown, Code 5: Distinct cavity with visible dentin, Code 6: Extensive distinct cavity with visible dentin) were annotated as caries cases, which may be closely related to the necessity of dental treatment clinically [23, 24].

### Deep learning algorithm application for tooth surface segmentation caries detection

Among the 2348 intraoral photographic images obtained from 445 patients during actual dental treatment, 998 images showing caries symptoms and 1350 healthy teeth without caries symptoms were divided. The number of local carious lesions found in the 998 images where caries was observed was 1999. This dataset is divided into three subsets: training, validation, and testing. 150 carious images corresponding to 15% of about 1000 carious images were first assigned to the test set, and the remainder were divided into a training set and an evaluation set at a ratio of 4:1. Among the collected data, since the number of normal images is larger than that of caries images, the number of test sets was set to 150, which is the same as that of caries images, to balance the classification model evaluation. For the remaining data, the training set and the evaluation set were divided at a ratio of 4:1. In the end, the dataset contained 1638 (69.8%), 410 (17.5%), and 300 (12.8%) images for training, validation, and testing (Table 1). Both the caries image and the normal image are used in the dataset for training or evaluation of the classification model and the segmentation model, and only the caries-containing image is used for the caries detection model.

**Table 1:** Dataset composition of intraoral photographic images

Subset	Photographic image			Carious lesions
	Total	Caries	Normal	
Train	1638	678	960	1468
Validation	410	170	240	368
Test	300	150	150	163
Total	2348	998	1350	1999

**Fig. 1:**
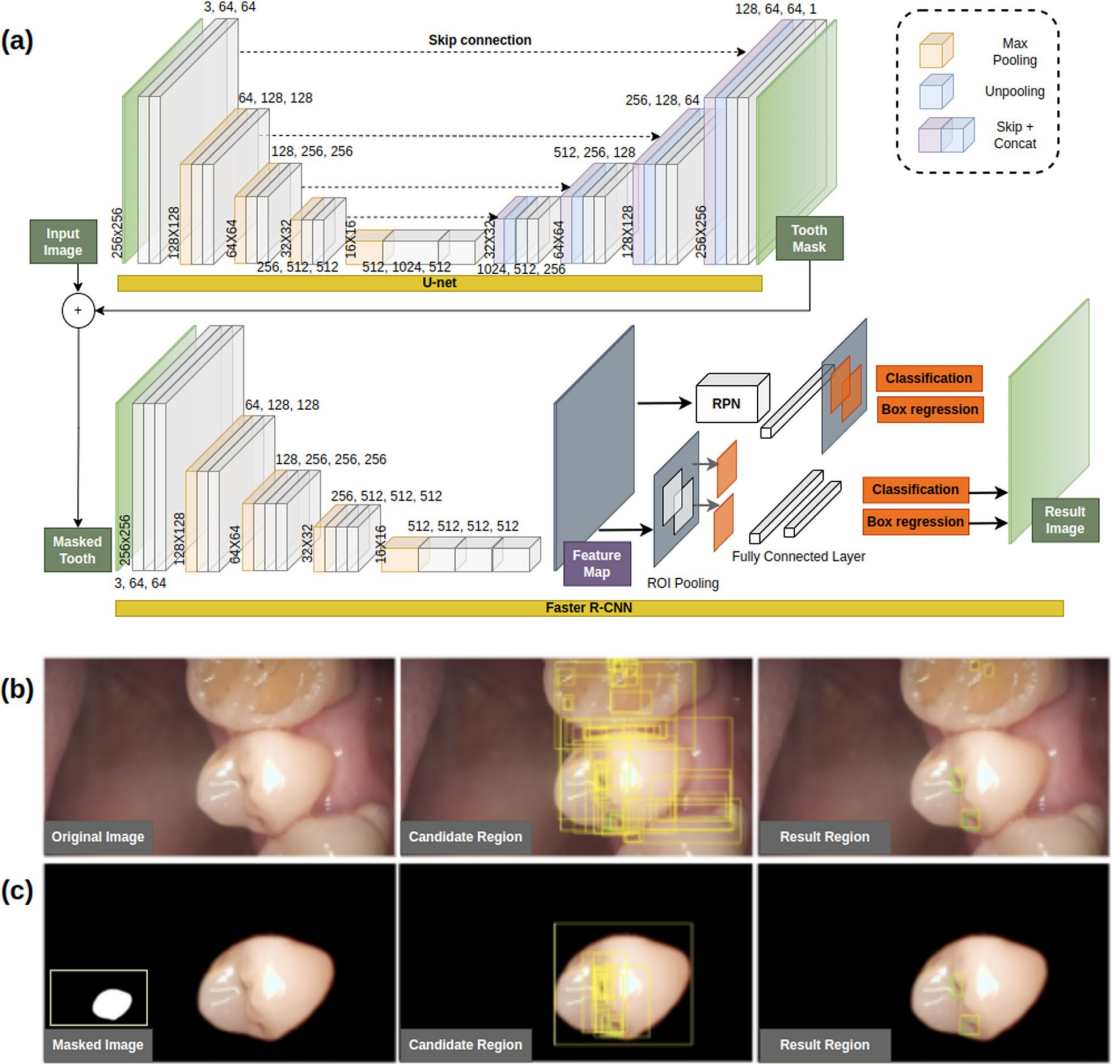
The architecture of U-net and Faster R-CNN for caries detection in IOC images. **a** U-net finds the tooth area in the IOC images and removes the background. U-net can divide the area through the process of progressively compressing and reconstructing the input information, and the skip connection helps to maintain the morphological characteristics of the previous information. Faster R-CNN creates a feature map by compressing input information with a backbone network such as VGG, and can recommend a caries region with a predefined anchor size through RPN (Region Proposal Network). Finally, we detect caries through a fully connected layer to the recommended location. **b** Images that have not been segmented increase false alarms in the RPN stage due to background noise. **c** After segmentation, it can be seen that the caries detection performance in the IOC has changed much more reliably

Images assigned to the testing subset of the CNN model were never used in the training or validation step for the independent evaluation of the deep learning model. To predict the tooth surface, U-Net was used, which is a CNN for biomedical image segmentation and can be used to extract objects from images [25]. Segmentation of the tooth surface improves the overall caries detection performance by darkening areas not classified as tooth surfaces in each image. ResNet-18 and Faster R-CNN were used for classification and localization of carious lesions, respectively. Figure 1 shows the caries detection structure using U-net and Faster R-CNN in IOC images. Through the U-Net segmentation model, a single tooth is targeted and the output of the RPN of Faster R-CNN is concentrated, so that false alarms are reduced compared to when the background is not removed through segmentation. ResNet is a CNN network that won the ImageNet Large-Scale Visual Recognition Challenge (ISVRC) in 2015. They proposed residual learning using skip connections and showed that the performance improves as the layer deepens compared to previous plain networks, such as AlexNet and VGGNet [26]. To check the usefulness of the deep learning technique in IOC data, we checked the general performance of the latest deep learning technique in caries classification using the ResNet-18 structure, which has the simplest structure among the ResNets.

Faster R-CNN is a deep learning algorithm that dramatically reduces the cost of the entire process by sharing the convolutional features of the classification stage with the region proposal network (RPN), which predicts the scores for the boundaries and locations of objects. We used Faster R-CNN to apply a state-of-the-art technique, which is commonly used in the field of object detection, for the location detection of caries [27].

### Training strategy of deep learning model

ResNet and Faster R-CNN were fine-tuned using pre-trained parameters with ImageNet and COCO train 2017 datasets, respectively. Besides, image augmentation, such as data shifting, image symmetry, and blurring, was applied to improve the generalisation performance of the model, but data generated owing to medical image compliance were not saved. For training, k-fold cross-validation was applied, and learning was performed by sequentially replacing separate validation sets of a certain ratio. The ratio of the training set to the validation set was 4:1, and 5-fold validation was performed on an average of 15–20 epochs; whether the validation loss increased by applying early stopping was used as the stopping criterion.

The selection of a loss function in deep learning model training can vary depending on which problem is to be solved. Ren, Shaoqing, et al. [27] used a multi-task loss function synthesizing classification loss and regression loss to simultaneously learn information on object presence and location in a Faster R-CNN model, and Mehdi Khoshboresh-Masouleh, et al. [28] optimizes the model more than before using weighted cross entropy to improve the result in the case of imbalance between the number of target pixels and non-target pixels in the segmentation problem. We used binary cross entropy and soft L1 loss by referring to the general method of Faster R-CNN. Binary cross-entropy loss was used for classification task between caries and non-caries in the Faster R-CNN model and each of predicted probabilities was compared with the ground truth either 0 or 1. Smooth L1 loss was used for box regression to estimate the location of caries. The loss function is defined as

1 $$L\left({p}_{i}, {t}_{i}\right) = \frac{1}{{N}_{cls}}{\sum }_{i}^{ }{L}_{cls}\left(p, {p}_{i}^{*}\right) + \lambda \frac{1}{{N}_{reg}}{\sum }_{i}^{{P}_{i}^{*}}{L}_{reg}\left({t}_{i}, {t}_{i}^{*}\right)$$

where ${N}_{cls}$ and ${N}_{reg}$ are the mini-batch size and the number of anchor locations, respectively. The anchor is a location information predefined as a candidate of the bounding box at each location of the image. $i$is the index of an anchor in a mini-batch and ${p}_{i}$ is the predicted probability of anchor $i$being an object, ${p}_{i}^{*}$is the ground truth, $t$is the vector representation of the bounding box, ${t}_{i}$ is the predicted box coordinate, and ${t}_{i}^{*}$ is the ground truth. The classification loss Lcls and regression loss Lreg are balanced by λ balancing parameter to prevent imbalances caused by different normalization factors between Ncls and Nreg. λ is normally and empirically set by 10 as default value. The classification loss Lcls is the binary cross entropy loss and the regression loss Lreg uses the smooth L1 loss function. The classification loss function is defined as

2 $${L}_{cls}\left({p}_{i}, {p}_{i}^{*}\right) = -{p}_{i}^{*}\text{log}{p}_{i} - \left(1-{p}_{i}^{*}\right)\text{log}\left(1-{p}_{i}\right)$$

The IOU (Intersection over Union) threshold was set to 0.2, and NMS (non-maximum suppression) was used to select the most suitable candidate area. The threshold value was set lower than for general object detection tasks; however, owing to the characteristics of caries, the boundary of the shape was not clear, and when compared with the ground truth, a false positive case occurred that was determined retrospectively to be correct. Thus, position errors were allowed to some extent. The SGD optimiser was used to update the parameters, and the learning rate was set to 0.001. In the case of ResNet, a dropout of 0.5 was applied, and the batch size was set as large as possible as memory allowed. For model training, two NVIDIA RTX2080 Ti GPUs and Itel Xeon E5-1650 CPU were used, and the deep learning frameworks, PyTorch 1.11, Cuda 11.4, and cuDNN 8.2, were used. Our code and benchmarks are available at github.com/hyeonrae/ioai.

### Statistical analysis

To evaluate the overall performance for caries classification, the accuracy, sensitivity, specificity, negative predictive value (NPV), precision, and area under the receiver operating characteristic curve (AUC) were calculated according to the segmentation of the tooth surface.

In the case of localization of caries, it is difficult to accurately identify a carious lesion in a photographic image in many cases even with a dental examination, and it is difficult to define a clear feature on the boundary between a carious area and a normal tooth area even on image data. It becomes difficult to precisely define the threshold of the boundary. Therefore, the allowable IOU threshold between the ground truth (GT) and the predicted bounding box was defined as 20%, and it was set more generously than other object detection models. If the IOU value was greater than 40%, the predicted box was regarded as the true detection case. True positive (TP), false positive (FP), and false negative (FN) cases were classified on this basis. To evaluate the overall performance of caries localisation, sensitivity, specificity, precision, NPV, precision, and average precision were calculated according to segmentation of the tooth surface.

## Results

The evaluation metrics of the classification algorithms for caries among 300 test images (150 images with carious teeth, 150 images of healthy teeth, with 163 carious lesions included) are shown in Table 2.

**Table 2:** Evaluation metrics of classification for caries images using test set

Evaluation metrics	Image segmentation for tooth surface	
	No	Yes
True positive case (n (%))	109 (36.3)	111 (37.0)
False positive case (n (%))	31 (10.3)	17 (5.7)
False negative case (n (%))	41 (13.7)	39 (13.0)
True negative case (n (%))	119 (39.7)	133 (44.3)
Sensitivity	0.722	0.740
Specificity	0.793	0.887
Negative predictive value	0.739	0.773
Precision	0.779	0.867
Accuracy	0.758	0.813
AUC	0.731	0.837

*AUC* area under the receiver operating characteristic curve

When image classification was performed with segmentation of the tooth surface, all the evaluation indexes improved better than those without segmentation. For example, AUC improved to 0.831 from 0.731. The accuracy improved to 0.813 from 0.756, which means that our CNN algorithms correctly classified 244 of the 300 test images of caries existence. The sensitivity and precision improved from 0.740 to 0.867 to 0.722 and 0.779, respectively. Pre-processing of each photographic image for tooth surface segmentation also improved the performance of CNN algorithms for the localisation of carious lesions. The sensitivity, precision, and average precision improved from 0.890, 0.874, and 0.889 to 0.865, 0.750, and 0.868, respectively, through segmentation of the tooth surface (Table 3).

**Table 3:** Evaluation metrics of localisation for caries before and after tooth surface segmentation using test set

Measure	Image segmentation for tooth surface	
	No	Yes
True positive case (n (%))	141 (67.1)	145 (78.4)
False positive case (n (%))	47 (22.4)	22 (11.9)
False negative case (n (%))	22 (10.5)	18 (9.7)
Precision	0.750	0.874
Sensitivity	0.865	0.890
Average precision	0.868	0.889

**Fig. 2:**
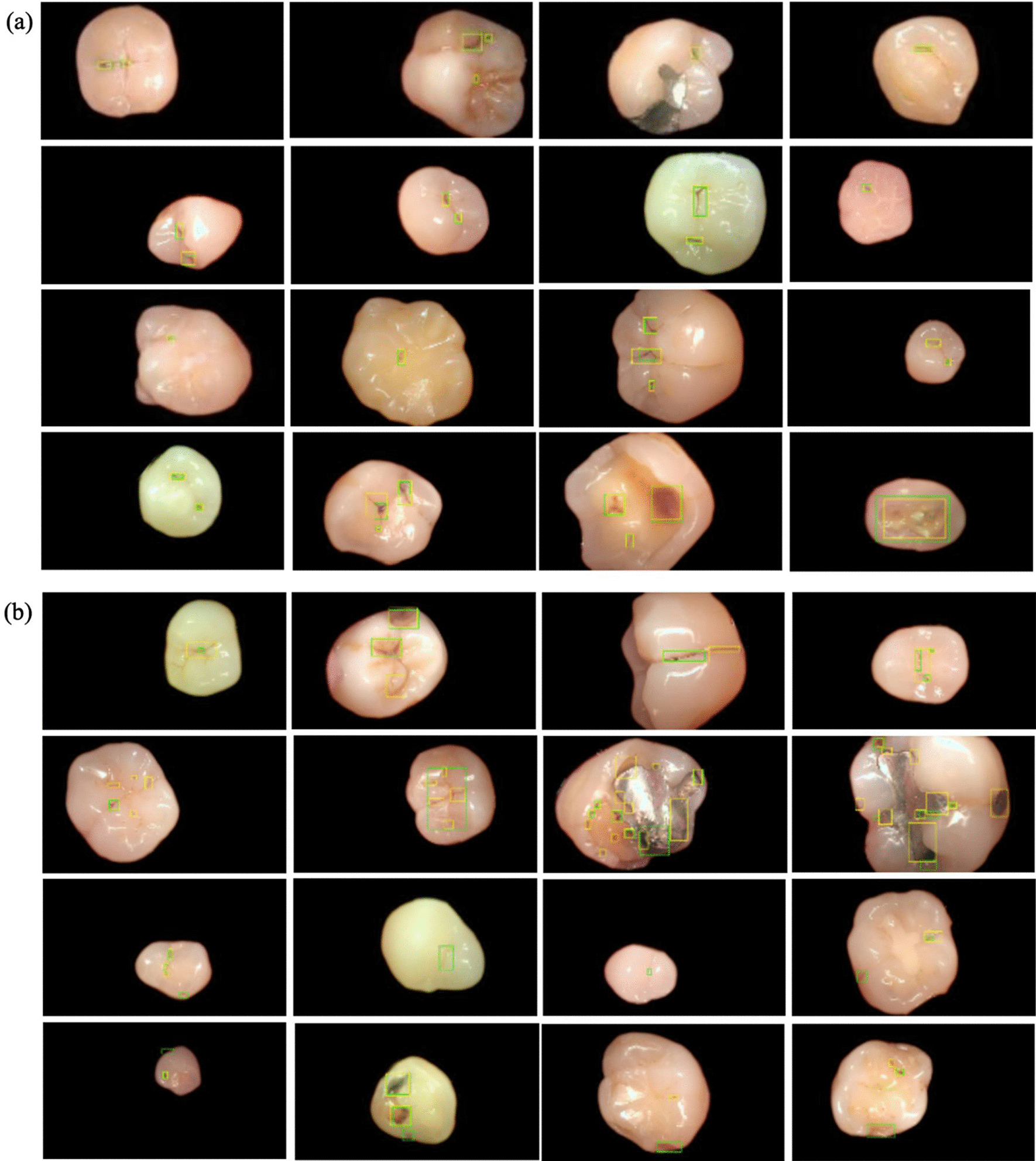
Results images. The green box is the ground truth cases and the yellow box is the predicted results by Faster R-CNN. **a** True positive (TP) cases when the IoU threshold is 0.2 **b** The results of false positive (FP) and false negative (FN)

From these results, we found that training CNN algorithms to predict the tooth surface in each photographic image can improve its performance in terms of both tooth classification and localisation of carious lesions. The detection results are shown in Fig. 2 for qualitative verification, in which the green box, GT annotated by the dentist and yellow box is predicted by the CNN model. In the TP cases, caries was appropriately detected, although the size of the bounding box may be slightly different from that of the GT (Fig. 2a). In many cases of false detection, they were classified as negative because there was a difference between the GT and prediction box. However, in such cases, clinical effectiveness could be expected because it properly included a significant proportion of the caries.

## Discussion

AI has recently proven promising in the detection and diagnosis of dental caries. Meanwhile, diagnostic imaging with AI in dentistry could operate much faster and more accurately than humans do, which lowers costs, eliminates subjective individual examinations, and increases the effectiveness of care [29]. However, most studies have applied deep neural networks to the analysis of dental X-ray images, such as panorama, bitewing, or periapical radiographs to detect dental caries [17, 30–32]. In clinical practice, visual-tactile examination is usually performed to detect caries as the first method. The caries detection method using intraoral photographs was more accurate than the inspection method, probably due to the ability to magnify the image on the computer monitor and thus easy to find [33]. Intraoral photographs can be beneficial for conducting dental public health surveys, especially in suburban areas. It could be utilised as an effective medium of communication between the patient and dentist [8] and in advancing tele-dentistry [34]. Intraoral photographs may be captured by a smartphone, professional single-reflex lens camera with flash, or intraoral camera for clinical use. Because smartphones have limitations in the magnification of the tooth surface, they are not recommended for identification of initial caries. In contrast, a professional single-reflex lens camera with flash is excellent in magnification and finding initial caries, but it is heavy, inconvenient to operate, and requires an intraoral mirror for the molar teeth. Therefore, intraoral cameras are generally used in dental clinics.

Therefore, in this study, we evaluated the performance of caries detection using a deep learning algorithm with photographs captured by dentists with intra oral camera for the classification of caries images and localisation of carious lesions. To improve the performance, we pre-processed the original images with a deep learning algorithm for segmentation of the tooth surface, which can extract the tooth image by eliminating the background from the original image automatically. As a result, all the performance indices, such as accuracy and AUC of the classification algorithm for caries images, were improved through segmentation of the tooth area. In the case of the localisation algorithm for carious lesions, improvement of the performance index, such as mean precision, was observed through segmentation of the tooth area. The detection system using Faster R-CNN is processed at an average speed of 270 ms per input image of 1280 × 720 size on the GPU (RTX2080), and the results can be utilized in real time by shooting with a camera in clinical practice.

In case of carious lesion detection, our model showed 89.0% of sensitivity and 87.4% of precision, which are better than 64.6% of sensitivity of previous study using oral photographs captured by smartphone [19]. However, in case of carious image classification, our model showed 83.7% of AUC, which is similar to 85.65% of the previous study [19]. According to another previous study on classification of caries image captured by DSLR, a high accuracy of 92.5% was achieved, which is better than 81.3% of our model [20]. However, as discussed in their paper, strict methodological requirements such as only single-tooth included image that did not have non-carious cases, for example, developmental defects, fillings, or discoloration were applied at their dataset, which cause potential problems in its application in general dental clinics [20].Therefore direct comparison of their performance with those of our CNN model is unsuitable considering that our dataset including various dental restorations, multiple teeth in one image, discoloration on occlusal tooth surface, which may have bad influence on prediction of CNN model. There are several drawbacks to intraoral photographs taken in dental clinics, such as presence of saliva, multiple teeth in one photographic image, images obliquely captured to the tooth surface, presence of dental restorations (such as amalgam and resin) or various lesions similar to caries. Therefore, this study attempted to improve the applicability of the DL model by reducing the limitations of the photography dataset and including various caries-like lesions for training.

However, most CNN models may show reduced performance when applied to real-world images with blur and noise because the neural networks are susceptible to these distortions. Therefore, most DL models may need to be trained using augmented images processed for various distortion [35]. Nevertheless, since augmented images are highly correlated with the original versions, these augmented versions do not provide many new features for the CNN to learn compared with independent original images. Therefore, we attempted to include various forms of oral photographic images as a dataset [36]. Besides, we pre-processed the original images through the segmentation of the tooth surface.

After our CNN model was trained for segmentation of the tooth surface, the resulting images included the tooth surface only by removing the background from the original images without manual intervention. Training the DL model for caries detection using these pre-processed images showed improved model performance compared to training using the original photographic images without pre-processing. Similar results have been observed in previous studies [21, 37–40]. In a study by Lian et al. a DL model showed good performance in detection and classification by localising the carious lesion after segmenting only the tooth outline within the entire image of the dental panorama [21].

Automated segmentation attempts using DL models have mainly been performed on radiography in dentistry, and this study is the first to be performed on intraoral photographic images. It is reasonable that segmentation of the tooth surface which removes the background from the original image and leaves only the tooth area, would contribute to improved accuracy by allowing the AI model to focus only on the tooth surface.

However, this study had several limitations. First, compared with an X-ray image, intraoral photographic images cannot express the inside of the tooth and the interproximal tooth surface. Therefore, there is a limit to fundamentally finding all carious lesions without X-ray images or tactile examination. In future studies, other diagnostic tools to complement the intraoral photographic images should be used as a dataset to evaluate the performance of the AI model. Second, the relatively low sensitivity and negative predictive value need to be improved along with the general improvement of all the evaluation indexes.

Nevertheless, this study is significant as it is the first report to achieve relatively acceptable performance of deep learning models in both classification of caries images and localisation of carious lesions using photographic images by intraoral camera. Diagnosis may vary depending on the clinician’s experience and skills therefore, CAD can be an effective diagnostic support tool to overcome this [41]. This study is expected to initiate the evaluation of the DL model performance using intraoral photographs and active investigations to diagnose caries easily and accurately.

## Conclusion

In conclusion, this study indicate that caries can be detected to some extent, even on intraoral photographic images, and this study can be developed to support the judgment of the medical staff.

## Data Availability

The datasets generated and/or analysed during the current study are not publicly available due to privacy of participants but are available from the corresponding author on reasonable request.

## Abbreviations

CNN: Convolutional neural networks
IOC: Intraoral camera
DL: Deep learning
AI: Artificial intelligence
CAD: Computer-aided diagnostic
DSLR: Digital single-lens reflex
STARD: Standards for reporting of diagnostic accuracy studies
ICDAS: International caries detection and assessment system
ISVRC: ImageNet large-scale visual recognition challenge
RPN: Region proposal network
IOU: Intersection over union
NMS: Non-maximum suppression
NPV: Negative predictive value
AUC: Area under the receiver operating characteristic curve
TP: True positive
FP: False positive
FN: False negative
GT: Ground truth
